# Challenges of Clinical Trial Design for Targeted Agents Against Pediatric Leukemias

**DOI:** 10.3389/fonc.2014.00374

**Published:** 2015-01-06

**Authors:** Francis Jay Mussai, Christina Yap, Christopher Mitchell, Pamela Kearns

**Affiliations:** ^1^School of Cancer Sciences, University of Birmingham, Birmingham, UK; ^2^Cancer Research UK Clinical Trials Unit, School of Cancer Sciences, University of Birmingham, Birmingham, UK; ^3^Department of Paediatric Oncology, John Radcliffe Hospital, University of Oxford, Oxford, UK

**Keywords:** pediatric, trial, leukemia, targeted, phase

## Abstract

The past 40 years have seen significant improvements in both event-free and overall survival for children with acute lymphoblastic and acute myeloid leukemia (ALL and AML, respectively). Serial national and international clinical trials have optimized the use of conventional chemotherapeutic drugs and, along with improvements in supportive care that have enabled the delivery of more intensive regimens, have been responsible for the major improvements in patient outcome seen over the past few decades. However, the benefits of dose intensification have likely now been maximized, and over the same period, the identification of new cytotoxic drugs has been limited. Therefore, challenges remain if survival is to be improved further. In pediatric ALL, 5-year-survival rates of over 85% have been achieved with risk-stratified therapy, but a notable minority of patients will still not be cured. In pediatric AML, different challenges remain. A slower improvement in overall survival has taken place in this patient population. Despite the obvious morphological heterogeneity of AML blasts, biological stratification is comparatively limited, and translation into risk-stratified therapeutic approaches has only best characterized by the use of retinoic acid for *t*(15;17)-positive AML. Even where prognostic markers have been identified, limited therapeutic options or multi-drug resistance of AML blasts has limited the impact on patient benefit. For both, the acute morbidities of current treatment remain significant and may be life-threatening alone. In addition, the Childhood Cancer Survivor Study (CCSS) highlighted many leukemia survivors develop one or more chronic medical conditions attributable to treatment (1, 2). As the biology of leukemogenesis has become better understood, key molecules and intracellular pathways have been identified that offer the possibility of targeting directly the leukemia cells while sparing normal cells. Consequently, there is now a drive to develop novel leukemia-specific or “targeted” therapies. These new classes of drugs will have mechanisms of action, toxicities, and therapeutic indices quite different from conventional cytotoxic drugs previously encountered, thus rendering current clinical trial methodologies inappropriate. Clinical trial methods will need to be adapted to accommodate these features of these new classes of drugs. This review will address the challenges and some of the techniques for developing clinical trials for targeted therapies.

## Conventional Versus Targeted Chemotherapy – What’s the Difference?

For both ALL and AML, despite the diverse range of pharmacological drug classes in use (e.g. DNA alkylators, topoisomerase inhibitors, antibiotics, steroids) treatment fundamentally revolves around drugs which are cytotoxic, causing intracellular damage that results in the death of leukemia blasts. The pre-clinical rationale for these agents is determined through cytotoxicity assays *ex vivo* and *in vivo* against whole populations of ALL or AML cells. Side-effects of treatment may be substantial, including severe myelosuppression, gastrointestinal, and neurological toxicity because of the non-selective mechanisms of cell killing. To manage these toxicities, conventional chemotherapy is frequently administered in cycles with gaps where no treatment is delivered, so as to allow recovery of healthy tissue cell numbers and function. Broadly, a successful drug leads to the eradication or substantial reduction in leukemia cell burden to a state of minimal residual disease (during therapy) or complete remission (CR) (at the end of therapy).

Different principles apply for targeted therapies: targeted therapies are aimed at pathways that function predominantly in leukemic cells, ideally with absent or minimal function in healthy tissues. Such pathways may be initiated by cell surface receptors (CD33, CD22), specific intracellular kinases (FLT-3; BCR-ABL); proteins regulating cell death (bcl-2 family), and modulators of gene expression (histone demethylases). In contrast to conventional cytotoxics, which are screened in whole cell assays, these agents may initially be identified using target molecule libraries or by rational molecular engineering, based on understanding of target molecule structure.

In turn, levels of target molecules or pathway function may be measurable in leukemia cells as biomarkers for patient selection or disease response. The ability to measure leukemia response to targeted therapy is particularly important, as binding of a targeted drug to its intended molecule may not directly induce cell death but cause other changes in cell function (e.g. maturation with retinoic acid in PML-RaR AML) or be cytostatic. As a result, the best dose-scheduling may not be determined by leukemia cell death but by efficacy against the target molecule – an optimal biological dose (OBD). Daily uninterrupted dosing over long time periods, instead of traditional “cycles” might prove to be the most appropriate method of administration, thus presenting further challenges to traditional early phase trial designs.

## Designing Clinical Trials for Targeted Agents

Clinical trials of drugs for any malignancy including leukemia have followed the traditional phase I, II, and III approach in which the primary outcomes of toxicity, disease response, and improvements in survival, respectively, are evaluated. In this section, the challenges of using this pathway to evaluate novel targeted therapies will be examined.

## Identifying a Safe Dose

Phase I dose-finding trials are performed following the completion of pre-clinical investigations, to identify a tolerable dose, which can be taken forward into a phase II trial to determine drug activity. The process has been based on the assumption of monotonic increasing dose–toxicity and dose–activity relationships. The primary objective of phase I trials is to identify the maximum tolerated dose (MTD), which is the highest safe dose that is believed to be the most efficacious for eradication of malignant cells. For hematological malignancies, phase I studies are conventionally performed on patients with relapsed or refractory childhood leukemias for whom there are no alternative therapies that might lead to cure. The rarity of the disease and the success of primary treatment protocols ensure that the numbers of patients eligible for phase I studies is extremely small.

The starting dose is pediatric phase I trials is often based on adult data, with 80% of the adult phase II dose, a commonly used starting point. Often, the dose levels to be evaluated are pre-specified. Doses could be escalated, with a 30% increase over the prior dose level, as a typical increment. In some cases, the dose escalation may continue beyond the adult phase II dose because of the different pharmacokinetics and safety profile in children (3). Toxicity within trials is assessed according to predefined systems such as the NCI CTCAE Version 4.0.

There are two main classes of phase I dose-finding designs, namely, the rule-based algorithms such as the 3 + 3 and its variations (3, 4), which include rolling-6 and the accelerated titration design, and the model-based designs. In the conventional and most widely used traditional 3 + 3 design, patients start at the lowest dose levels based on pre-clinical or adult data, dependant on the trial age group, in cohorts of three patients. If no patients experience dose-limiting toxicity (DLT) at that drug dose, the next cohort will be given the next higher dose. If a DLT is observed in one out of three patients, three additional patients will be treated at the current dose. The MTD is defined as the highest dose level where fewer than two out of six patients experience DLT.

In the “rolling six” design, enrollment may continue even when three patients have entered a dose cohort (4). If there is no DLT in the first three patients, then the fourth patient can be allocated to the next higher dose level. If toxicity data are not available on the first three patients, or if one DLT is observed, in which case the new patient would be kept at the same dose level. If two or more DLTs are observed in a single cohort, the dose level is decreased. The main advantage of the design over the 3 + 3 design is the reduction of the overall trial duration as it aims to decrease the number of times the trial is suspended to accrual (4, 5). However, this trial design may also lead to more patients than necessary being recruited at lower, perhaps inefficacious, dose levels than a 3 + 3 design. An alternative to the rule-based algorithms are the model-based designs, such as the continual reassessment method (CRM). Data accrued are continuously used to predict the dose level at which DLT is likely to be reached, based on Bayesian or likelihood modeling for the CRM (6, 7). The MTD is defined as the dose level with a pre-determined toxicity probability, usually in the 15–40% range. Although recently utilized in adult hematology drug development trials, the use of the CRM design has not been extended to pediatric leukemia studies (8). One example of the application of CRM is in a study to evaluate a targeted anti-apoptosis inhibitor (navitoclax, BH3 mimetic, and BCl2 inhibitor) in adult chronic lymphoblastic leukemia (CLL). The study used a small population of patients (*n* = 29), analogous to typical pediatric studies, and the drug was given daily over a relatively long period of time (median 7 months, range 1– >29 months). This study illustrates how pediatric trial consortia could develop this model (9). A new radionucleotide therapy for pediatric osteosarcoma (Samarium-153-EDTMP) has already been tested using this trial design (10). The Pediatric Brain Tumor Consortium has also used a modified CRM to evaluate a gamma-secretase inhibitor (NOTCH pathway) in CNS tumors (11). Despite higher statistical complexities, such model-based designs have demonstrated superior operating characteristics in terms of correctly identifying the right dose to take forward to a phase II trial and allocating more patients to the MTD than rule-based 3 + 3 designs (12, 13). Moreover, the sample size required for such designs to have a satisfactory performance is usually less than the maximum sample size that is required for a 3 + 3 design. However, rule-based designs are still very appealing as they are very simple, easy to implement in practice, and do not require any specialist software or programing. Hence, they are still commonly used in practice. Nevertheless, in recent years, model-based adaptive designs are gaining in popularity as researchers are made more aware of their advantages and there is increasing availability of software to implement such designs.

Both the rule-based algorithms and model-based designs described so far assume that as the dose of drug is increased, the activity (i.e., effect on leukemia cell death) increases, and there will be a corresponding increase in toxicity to healthy tissues. As such, toxicity is judged to act as a correlate for drug effect. This assumption is reasonable for conventional cytotoxic chemotherapy in pediatric leukemias. A retrospective analysis of 85 children treated for ALL at a single center on MRC UKALL protocols between 1973 and 1990, revealed an 18% improvement in relapse-free survival when the dose of 6-MP was increased toward the 6-MP MTD, and dose increases on average of 22% were achieved (14). Analyses of POG studies (POG 8101, 8498, 8821, 9421) have similarly shown that increasing the dose intensification of cytarabine toward the MTD results in improvement in survival for children with AML (15). For targeted agents, however, the relationship between target effect and toxicity may not be linear, and hence require different strategies for phase I studies. Targeted agents may be essentially safe within the therapeutic dose range. They do not induce toxicity in healthy tissues and hence the effects on non-malignant tissues cannot be used as a marker of drug efficacy. Furthermore, targeted agents may have a plateau in effect, meaning a higher dose will not improve patient benefit, although it might result in toxicity. Therefore, the aim of the trial is not to identify the MTD, but the OBD or most desirable dose, which can be defined as the most favorable dose with acceptable toxicity and high efficacy. This requires the model to not only select the most appropriate dose based solely on toxicity but also to examine both toxicity and activity.

Though there is an increasing use of targeted agents in drug development in oncology, there are very limited statistical models that have been developed to utilize both toxicity and activity to obtain OBD. The pioneers in this field include Thall and Cook who developed EffTox, Zhang, Sargent, and Mandrekar who proposed an extension of the CRM-TriCRM, and a recent paper by Hoering et al. on the use of a 3-arm modified selection design (16–18). Very few of such designs have been implemented in practice, partly due to difficulty in defining a suitable activity endpoint for assessing targeted agents (which will be discussed later), as well as the complexity of such designs (19). Others have proposed more efficient models to measure overall severity of multiple types and grades of adverse events, after considering that targeted agents tend to induce multiple moderate toxicities rather than a binary end point of DLT that is used conventionally (20).

Data from a number of adult and pediatric trials illustrate that even a well engineered targeted agent may exhibit “off-target” toxicity for a number reasons including non-specific binding, pathway homology, and previously unknown expression of the target molecule in healthy tissues.

Examples of such unexpected toxicity are found for all classes of novel targeted agents. Gemtuzumab ozogamicin (GO), is an anti-CD33 antibody conjugated to calicheamicin toxin and was given accelerated FDA approval for the treatment of relapsed AML in older patients not suitable for more intensive therapy. GO became one of the few newly licensed therapies for AML in decades and was assumed to offer a high degree of specificity since CD33 expression is limited to myeloid cells. However, in 2010, the drug was voluntarily withdrawn because clinical trials suggested a lack of improvement in overall survival and an excess of toxicity, including veno-occlusive disease, which was unexpected based on the molecule’s pre-clinical history and known mechanism of action (15). Unexpected “off-target” toxicity has also been experienced with another immunotherapy, engineered T cells. Here, T cells designed to have high affinity for a MAGE-A3 epitope expressed on myeloma and melanoma cells were found to cross-react with an epitope on the protein titin, leading to fatal cardiomyopathy (21). Previous studies and pre-clinical investigations had not predicted the likelihood of this toxicity (22, 23). Small molecule enzyme inhibitors have also demonstrated unexpected toxicities. Experience with tyrosine-kinase inhibitors (TKIs) illustrate that toxicities cannot be assumed to be similar even if structurally related drugs have been rationally derived. The small molecule TKI sunitinib (c-kit, flt-3, VEGFR2, PDGFR-beta inhibition) is noted to have cardiac toxicity as a DLT in a pediatric solid tumor phase I study, while sorafenib (c-kit, flt-3, VEGFR2, PDGFR-beta, and RaF-pathway inhibition) has completed phase I testing in children with leukemia without this toxicity (24, 25). Therefore, investigators should be aware that toxicities from novel targeted agents may be unexpected and more varied, compared to conventional chemotherapy.

An alternative approach for appraisal of novel agents may be to incorporate phase 0 studies into the drug development pathway. These studies expose a small number of patients to a low dose and duration of the drug and provide limited data on toxicity and bioactivity. Although phase 0 studies have been performed successfully for targeted agents in adults with hematological malignancies, none have yet been reported for children with leukemias (26).

A further challenge provided by conventional phase I trial design is that toxicity is assessed in a relatively short time frame after drug administration. Although short-term toxicities are important, some targeted drugs are likely to be beneficial over a continuous, longer maintenance period, potentially for years, but no mechanism exists for assessing long-term toxicity within current early phase strategies. Trials, which allow dose escalation based on acute toxicity, may well underestimate or overlook significant dose-related side-effects that can occur after prolonged use of targeted agents, particularly in the pediatric population where physical growth and tissue maturation continues.

Alternative phase I trial designs that evaluate toxicities secondary to chronic use may be more relevant if the proposed use of the targeted agent is long-term. Time-to-event CRM, tite-CRM (27), offers an attractive advantage of allowing for late onset toxicity to guide dose escalation, allowing the DLT observational period to be much longer, for instance, for up to six treatment cycles. The main idea of the tite-CRM is to estimate the dose–toxicity relationship based on all the available DLT information, including those patients who might not have completed their full DLT observational period, to guide the dose for the next patient. This will avoid stopping the trial for a long period of time to assess DLT of the last recruited patient. CRM has not yet been used for novel agent trials in pediatric leukemias and could be more actively considered by investigators and clinical trial consortia.

There is a limit to how much safety monitoring can be achieved within the time constraint of a trial. Although short-term follow-up of early phase clinical trial patients is commonly included in protocol design (often 3–5 years) detailed long-term follow-up is not. Such “late-effect” studies are frequently separate studies or academically sponsored, and therefore, the long-term effects of exposure to targeted agents is generally unknown. As many drugs are tested on multiply relapsed patients who eventually die of disease within a relatively short time-span, the number of patients to accrue meaningful data from is small. For those that survive, knowing which data are of relevance with targeted agents to capture over the long-term is also unclear. Addressing this issue is difficult and requires concerted effort by drug companies, academia, and regulatory agencies to report data.

The TKIs (imatinib mesylate, dasatinib, nilotinib, and bosutinib) represent the paradigm of targeted therapies, in which imatinib was identified in high-throughput screens to be an effective inhibitor of ABL kinase activity (28). In the COG phase I trial of imatinib, eligible patients were treated with daily drug dosing for 28 day cycles (median 6 cycles; range 1–46 cycles) (29). The dose level was expanded to a maximum of six patients if one had DLT during the first course. The MTD was defined as the level immediately below the level at which two patients in a cohort of no more than six had DLT. The drug was tolerated with mostly grade 1 and 2 gastrointestinal toxicities. There was only one episode of first course DLT (grade 2 weight gain) and no MTD was defined. However, in contrast to the trial study period patients, particularly those with chronic myeloid leukemia, are treated with TKIs for years. One retrospective study, in which children received a median of 16.2 cycles of imatinib (range 31–83 cycles), far longer than the median toxicity evaluation in the original phase I trials, found significant growth retardation in pre-pubertal children.

Establishing a safe dose for targeted agents against pediatric leukemias faces a number of challenges; not least the limited experience of using trial methodology other than traditional 3 + 3 phase I design. Investigators may need to use alternative endpoints to toxicity, such as target inhibition or binding, drug concentrations in the microenvironment (blood or bone marrow pharmacokinetics) or biomarkers of activity (see next section) to determine drug dosing and to make critical decisions on further investigation of potentially active drugs.

## Measuring Biological Effect

Traditional pre-clinical testing has proposed therapeutic doses and schedule for phase I trials, which then determine the recommended phase II dose based on toxicity. In the era of targeted agents, a more appropriate end point would be the drug activity against the target site, i.e., the OBD. This concept encroaches on the traditional purpose of phase II studies. One challenge is therefore to consider if phase I and II studies would better be combined for targeted agents, so that a safe dose is rapidly identified but alongside assessment of biological effect. In this section, the concept of measuring effect on target pathways and phase II trial designs will be discussed.

The objective for phase II trials is disease response, for example, measured by clearance of blasts from the bone marrow, defined by established criteria (30). For hematological malignancies access to samples of malignant cells before, during, and after treatment is readily available, through collection of malignant cells from blood and bone marrow. Sensitive techniques are well-established and include flow cytometry and PCR-based techniques to detect even rare populations of leukemia cells. Therefore, for targeted agents which are cytotoxic, alterations in phase II design may not be needed. A recent phase II trial of the proteasome inhibitor, bortezomib, demonstrated an 80% CR and CR without platelet recovery (CR + CRp, respectively) response rate in children with advanced B-precursor ALL after a single course of treatment, with a significant increase in overall survival compared to those patients whose disease did not respond to this agent (31).

However, traditional cytotoxic endpoints may be invalid for other classes of novel targeted agents. Plerixafor is a small molecule antagonist to CXCR4 expressed on AML blasts, which prevents binding to chemo-protective stromal cells. In an adult phase I/II trial, no improvement in CR or overall survival was seen. However, a 2.5-fold increase in blast mobilization into the peripheral blood was found, confirming biological activity at the dose level tested (32). It is hypothesized that the liberation of cells into the peripheral blood might improve efficacy for other drugs since the cells would no longer enjoy the haven of the stromal protection. Hence, this drug will need to be studied in combination with other agents where dose sparing or increased efficacy might be achieved.

Further complexities exist when novel agents only target sub-populations of blasts. Mutations may occur at different points of blast evolution such that significant numbers of clones in a patient will remain unaffected by the targeted therapy. Adding to this, there is redundancy such that no single mutation is likely to be the only driver for AML blast development in all cells. Assessment of the target activity of such drugs may well require sub-group or single-cell assays in combination with genomic studies. Activating mutations of FLT3, a receptor tyrosine kinase, have been identified in AML and often occur late in AML development. FLT3 mutations have been targeted by a number of small molecule inhibitors. A (phase II) trial of lestaurtinib in combination with standard chemotherapy for patients with relapsed AML expressing FLT3-activating mutations had mixed outcomes (33). The majority of patients achieved biological target effect, as measured by target FLT3 inhibition. In turn, FLT3 inhibition correlated with CR and higher plasma drug levels; however, there was no significant improvement in remission rates or overall survival. Although high levels of drug toxicity but did not correlate with FLT3 inhibition *in vivo*. A number of other possible explanations exist for these mixed results including a failure to sustain plasma levels of study drug, or binding of drug to plasma proteins reducing the free, active drug. Therefore, even with patient selection to ensure relevant target exists, a full understanding of both the underlying biology and the pharmacodynamic interactions of the drug are needed in the interpretation of trial results. Instead of measuring cell death by morphological or molecular remission, “time-to”-endpoints, such as progression-free survival, time to hematopoietic stem cell transplant, or overall survival may be more suitable end points for evaluation of targeted agents. However, the question of how to design such studies with an appropriate control group to confirm study drug activity remains difficult. Many have argued that the use of historical control is only appropriate in settings when there is no standard treatment, presence of well-established historical control data (especially if it is recent), very low response rates in the standard treatment, or if the study population is very limited, which renders an extra arm of patients undergoing the control treatment to be infeasible. Otherwise, historical controls or hypothetical standardized survival times are often unreliable, and a randomized trial with a control arm is important to provide robust preliminary data to inform future phase III trials (34).

Several frequentist and Bayesian single and multi-stage phase II single arm designs have been developed for binary endpoints (35, 36). In single stage designs, a pre-specified drug response rate is evaluated on a pre-determined number of patients based on the required statistical power of the predicted effect. The disadvantage is that all subjects have to be treated before a conclusion about drug effect can be made, thus potentially exposing children to the unnecessary risk of ineffective new agents. For ethical reasons, Simon’s two-stage designs and comparable Bayesian’s multi-stage designs which allow for interim evaluation for futility to overcome limitations are commonly used (37). Here, the goal is to use a small number of patients to determine drug activity, and the trial is terminated early if activity criteria are not met. Such design offers the opportunity to prevent too many patients from exposure to an inactive study drug. As the number of novel agents grows this design could also allow the relatively small pool of relapsed/refractory leukemia patients to be eligible for a number of studies. Although one can evaluate time-to-event endpoints as a binary outcome, for example, progression-free survival at 6 months (simply as a proportion of all subjects who survive at the required time-point), this will, however, give a biased and an inefficient estimate of the survival probability if the follow-up periods are incomplete. Case and Morgan developed a more efficient two-stage design for evaluation of time-to-event outcomes, incorporating all the survival information up to the time-point of interest (38).

A further challenge in evaluating new targeted agents is the improbability that a single agent will be effective. In acute leukemia, leukemogenesis arises from multiple, overlapping, biological pathways. It seems likely, therefore, that blasts might be resistant to highly targeted agents. Further, because these drugs are being tested in heavily pre-treated patients, the question of whether the biology of leukemias in such patients is representative of leukemias in newly diagnosed patients arises, and further complicates the evaluation of efficacy.

Ideally, novel drugs would be tested in untreated patients, but the excellent cure rate for many leukemias and the desire not to delay established treatment makes this approach unpalatable. Indeed, the introduction of the new classes of drugs into routine first line therapy will require a major reappraisal of the concept and conduct of phase III studies, possibly including the notion that achieving the same cure rate but with less toxicity is a worthy goal.

In certain instances, for instance, very high-risk patients where the outcome using conventional therapies is poor, or where a clear, treatable target exists, such as BCR-ABL, phase II window studies may be a better approach, so that targeted agents would be assessed in an initial “window” before conventional treatment starts. To date, this notion has been little used in pediatric leukemias. A single example has been a phase II window study of single-agent rituximab for children with newly diagnosed B-non-Hodgkin’s lymphoma (B-NHL) (39). The study successfully evaluated the response rate of rituximab in chemo-naïve patients; importantly no increase in relapse rate was seen despite the delay in starting conventional therapy.

Phase II multi-arm strategies are an increasingly attractive, efficient approach to evaluating more than one novel treatment in a single trial. In these studies, patients are initially randomized between a control standard of care arm and a number of different novel drugs. All drugs are only evaluated against the control arm and not against each other. Multi-arm study design does not answer the challenges of selecting a suitable endpoint for novel agents but has a number of advantages. It is highly flexible and allows new drugs to be added at any time within the trial timeline by protocol amendment, and speeds up the number of drugs able to be tested in a limited pool of patients. The first part of the study ensures unpromising treatments are dropped, and those with potential efficacy continue to be tested. For a drug to proceed, it must show a pre-determined degree of efficacy against selected outcome measures (CR rates or surrogate markers) compared to control. The design incorporates a minimal, clinically relevant difference that is large between novel drug and control arm such that drugs that do not show activity are dropped early on. Promising agents can then be taken forward for full evaluation in a randomized controlled phase III trial, where the crucial question of whether the new agent brings additional benefit over standard therapy can be addressed. The design is also advantageous as a fixed number of control patients can be used to compare against patients on other arms, thus reducing the number of patients and trial costs required per study drug in comparison to two arm studies. This “Pick a Winner” approach has not been used for pediatric leukemia studies but has been used to good effect to test a number of new targeted agents for AML in an elderly adult population (40). Further design permutations have been statistically modeled including combining a Simon two-stage design followed by a play-the-winner approach (screened selection design, SSD), although not yet implemented into clinical practice (41).

There is also scope to further explore Bayesian methods to evaluate how information from adult leukemia trials can inform pediatric trials with appropriate use of pharmacodynamics and pharmacokinetic data in phase II trials. This is particularly useful, if there are already completed trials on the targeted agents in the same adult population. Methods to consider dynamic borrowing of historical adult data, where the amount of weight assigned to the historical adult data depends on how consistent it is with the observed trial data on children could be explored (42).

## Phase III Challenges

It could be said that current treatment strategies for both AML and ALL have evolved to the point where patients either have an excellent or a negligible chance of cure and that these two groups can be defined very early in the course of the disease and therapy, either by pretreatment factors such as cytogenetic of molecular genetic factors or by response to therapy as assessed by minimal residual disease. It has already been noted that patients with a poor chance of cure with current therapy might be better served by enrollment into phase II window studies, where early exposure to a novel drug might lead to improved efficacy compared to the conventional regimen or later exposure to the novel drug. A far greater challenge is the development of a phase III trial method that will allow incorporation of novel drugs into a conventional regimen, presumably substituting for one or more of the conventional drugs included in the standard regimen, since addition of a new agent to an already successful regimen – where EFS may well already be in excess of 95% – is unlikely to yield any improvement in cure rate. The goal in this setting would likely be equivalence, although a reduction in treatment related mortality might mean that experimental treatment group had, in fact, a better outcome than the conventional group. The problems here are the need for large numbers of patients, especially given that the multiplicity of biological factors means ever-increasing sub-groups of smaller size, and how to determine which of the conventional drugs should be omitted.

## Ethical Dilemmas in Targeted Agent Trials

The majority of early phase clinical trials for pediatric hematological malignancies take place on patients with multiple relapses or refractory disease. For researchers, trials are aimed at evaluating novel drug toxicity and efficacy. For patients, and particularly their families, the main driver for entry onto a drug trial is a hope that the study drug will contribute to a cure, even against all odds. Further complexity is added as research legislation in Europe and the US appropriately consider children as vulnerable patients who are dependent on a responsible adult to make decisions in their best interest. However, a number of studies have identified that parents with children enrolled on oncology trials have only a limited understanding of the research aims and the risks of treatment, but perceived that the trial drug would be of benefit in terms of their child’s probability of cure (43–45). Therefore, both standard and targeted agent trials share the significant challenge to explain adequately the purpose of early phase trials, which may offer no benefit to an individual patient or conversely induce toxicity with no anti-leukemia activity. Consenting doctors involved in the process of obtaining informed consent may also have conflicting roles as both the patient’s primary physician and study investigator.

## Conclusion

Although the development of intensive multi-agent chemotherapy regimens, with concomitant advances in supportive care have led to major improvements in outcome for children with acute leukemia, particularly of the lymphoid and also of the myeloid type, treatment related toxicity and mortality, together with a persisting problem of treatment failure in a small but significant minority of patients means that further developments in treatment are needed. It is likely that the major improvements in outcomes seen in the 1980s through to the early 2000s, arose as a consequence of dose intensification, and further improvements by this avenue will not be attained. Thus, the time is ripe for the introduction of new classes of “biological” agents. Current models for phase I and II studies may well not be appropriate for these types of drugs. Thus, the notion of the MTD and the need for a separate assessment of efficacy may be supplanted by a single study combining the identification of an OBD with confirmation of expected target pathway efficacy.

In the longer term, the need for better therapy of identifiable high-risk groups will necessitate the development of phase II window studies. These studies, together with the hybrid phase I/II OBD studies, may give some notion about which conventional drugs in a standard regimen can be substituted by novel drugs with at least equal efficacy but much improved safety. The ultimate goal should be the use of a rationally tailored combination of biological agents based on knowledge of the leukemogenic events for each individual patient. This approach will lead to some very novel and challenging problems in trial design, where sub-group sizes will be very small – perhaps only 10’s or even fewer patients – and the expected outcome will be that all patients are cured.

## Conflict of Interest Statement

The Review Editor Christian Michel Zwaan declares that, despite having collaborated with author Pamela Kearns, the review process was handled objectively and no conflict of interest exists. The authors declare that the research was conducted in the absence of any commercial or financial relationships that could be construed as a potential conflict of interest.
